# Impact of submacular fluid volume on visual outcome in macula‐off rhegmatogenous retinal detachment using automated optical coherence tomography volumetric quantification

**DOI:** 10.1111/ceo.13929

**Published:** 2021-05-05

**Authors:** Reinhard Angermann, Stefan Mosböck, Christoph Palme, Hanno Ulmer, Teresa Rauchegger, Yvonne Nowosielski, Nikolaos E. Bechrakis, Claus Zehetner

**Affiliations:** ^1^ Department of Ophthalmology Medical University Innsbruck Innsbruck Austria; ^2^ Department of Ophthalmology Paracelsus Medical University Salzburg Salzburg Austria; ^3^ Department of Medical Statistics, Informatics and Health Economics Medical University Innsbruck Innsbruck Austria; ^4^ Department of Ophthalmology University Hospital Essen Essen Germany

**Keywords:** macular detachment, retinal detachment, retinal imaging, visual acuity

## Abstract

**Background:**

We investigated effects of submacular fluid volume (SMFV) on visual outcomes following surgery for macula‐off primary rhegmatogenous retinal detachment (RRD) using automated fluid volumetric quantification with optical coherence tomography (OCT).

**Methods:**

We analysed 127 eyes that were surgically treated for macula‐off RRDs. We obtained preoperative images following the spectral domain (SD)‐OCT dense volume protocol, applied automated retinal segmentation and used an automated algorithm to quantify each eye's SMFV. We used multivariate models to identify various risk factors for impaired visual outcome.

**Results:**

Linear regression showed that preoperative SMFV (ß = 0.013; *P* = .005) was significantly associated with a reduced visual outcome 12 months after the treatment of macula‐off RRDs. SMFV was negatively correlated with 12‐month postoperative (*r* = .311; *P* = .001) visual acuity (VA). The group with low preoperative SMFV (≤9.0 mm^3^) showed an increasing VA up to 12 months postoperatively (*P* < .001), while the VA did not increase in the group with high SMFV (>9.0 mm^3^) beyond 3 months of follow‐up. Patients with a high SMFV were 8.0 times more likely to have worse visual outcomes after 12 months of follow‐up (*P =* .018).

**Conclusions:**

SMFV was negatively correlated with visual outcomes after the surgical treatment of macula‐off RRDs. Patients with SMFV <9.0 mm^3^ 12 months after surgery had an 8.0 times greater chance for better visual recovery than patients with high preoperative SMFV. Our findings highlight the efficacy of automated SMFV quantification in predicting surgical outcomes in patients with RRDs, which could be useful in future clinical practice and the development of research models.

## 1 INTRODUCTION

Macular detachment is a major negative prognostic indicator for visual acuity outcomes following surgery to treat primary rhegmatogenous retinal detachment (RRD). Even after surgical reattachment, in cases of central detachment vision improvement and neuroretinal recovery remain compromised because of permanent functional damage to the macula.^1^, ^2^ Optimal surgical timing remains debatable, a tolerable cut‐off that varies from 1 to 7 days has been proposed to have a beneficial effect on final visual acuity (FVA).^3^, ^4^, ^5^, ^6^, ^7^, ^8^ Other factors, such as age, preoperative visual acuity, proliferative vitreoretinopathy (PVR), the extent of detachment and operation procedure, were reported to have an additional impact on FVA.^9^, ^10^, ^11^ With the use of optical coherence tomography (OCT), cystoid macular oedema (CME) and macular detachment height have been added to the list of potential negative predictive factors.^12^, ^13^

Previous studies investigating the importance of oxygen and nutrition supply in experimental RRD have supported the association between detachment height and photoreceptor damage.^14^, ^15^ According to these studies, we hypothesised that along with detachment height, the area of detachment and subretinal fluid volume would affect oxygen diffusion from the choriocapillaris to the detached neurosensory retina. Furthermore, the effect of preoperative submacular fluid volume (SMFV) on surgical outcomes in RRD cases has not previously been investigated.

To address this research gap, we employed an automated method to quantify SMFV using the segmentation software designed for spectral‐domain (SD)‐OCT. The main objective of our study was to determine the effect of SMFV on the visual outcome in patients undergoing surgical treatment for macula‐off primary RRD by comprehensively analysing clinical, surgical and SD‐OCT findings.

## 2 METHODS

The data of all patients with primary RRD were collected from the Department of Ophthalmology of the Medical University of Innsbruck, Austria from a standardised structured electronic database. The implementation and application of the RRD register were validated by the Department for Strategic Quality Management of the University Clinic Innsbruck, Innsbruck, Austria. According to the dataset protocol, all patients underwent SD‐OCT (OCT Spectralis, Heidelberg, Germany) and ultra‐widefield retinal imaging.

Our retrospective register study was approved by the Local Committee for Medical Research Ethics. Due to the retrospective nature of the study, the requirement of consent was waived by the institutional review board of the Medical University of Innsbruck, Innsbruck, Austria. All procedures involving human participants were performed in accordance with the ethical standards of the institutional and/or national research committee and the 1964 Declaration of Helsinki, and its later amendments, or comparable ethical standards.

### 2.1 Setting

This institutional retrospective study was conducted from January 2014 to June 2019 at the University Clinic Innsbruck, Innsbruck, Austria.

### 2.2 Study population

We enrolled 127 consecutive patients with macula‐off RRD, all of whom received small‐gauge (G) vitrectomy (23G, 25G, 27G) for surgical reattachment. The data were collected using a structured electronic database for patients with macula‐off primary RRD. Included patients presented directly to the emergency department of the clinic or were referred from other hospitals or private ophthalmologists.

The inclusion criteria were: (a) patients with primary RRD without macular involvement, (b) patients with a follow‐up of at least 6 months after surgical reattachment and (c) patients with no complications. The exclusion criteria included patients with any of the following: (a) previous vitreoretinal surgery; (b) vitreous haemorrhage at first presentation; (c) re‐detachment during the follow up; or (d) a history of posterior uveitis, penetrating or non‐penetrating trauma, potential sight‐threatening diseases (significant cataracts, diabetic retinopathy, age‐related macular degeneration, glaucoma, amblyopia, or retinal vein occlusion) developed during the study or in the fellow eye.

The following data were collected for analysis: sex; eye affected; lens status; best‐corrected visual acuity (BCVA) displayed as the logarithm of the minimum angle of resolution (logMAR) before surgery as well as 3, 6 and 12 months after surgery; onset of central vision loss; the presence of pathological myopia (axial length ≥ 26.5 mm); a history of trauma and complicated surgeries (including cataract surgery, previous retinal surgery and laser therapy); the presence of vitreous bleeding; characteristics and location of retinal tears; the extent of retinal detachment and affected quadrants; the presence of PVR and the PVR grade; surgical technique (PPV gauge 23, 25 or 27); foveal detachment; and various OCT characteristics (maximum detachment height, central macular thickness [CMT] and SMFV at a diameter of 6 mm centred on the fovea). After the diagnosis, all patients were admitted to the ward for posturing while awaiting surgery.

### 2.3 Intervention/observation procedure

We used electronic medical records to analyse the patients' surgical outcomes. Experienced retinal specialists performed ophthalmological examinations while the patients were hospitalised. We assessed the foveal status on SD‐OCT images using a standardised protocol for RRD. To acquire the images, we used a dense volume scan protocol with 25 B‐scans, wherein each scan comprised 512 A‐scans and a field of view of 20° × 20° (6 × 6 mm). An eye‐tracking software (TruTrack, Heidelberg Engineering, Heidelberg, Germany) ensured automated eye alignment. The SD‐OCT software automatically executed retinal layer segmentation. For each case, we confirmed that foveal fixation and correct segmentation were adequate in the 25 B‐scans; a retinal specialist then manually corrected smaller segmentation failures.

**FIGURE 1:**
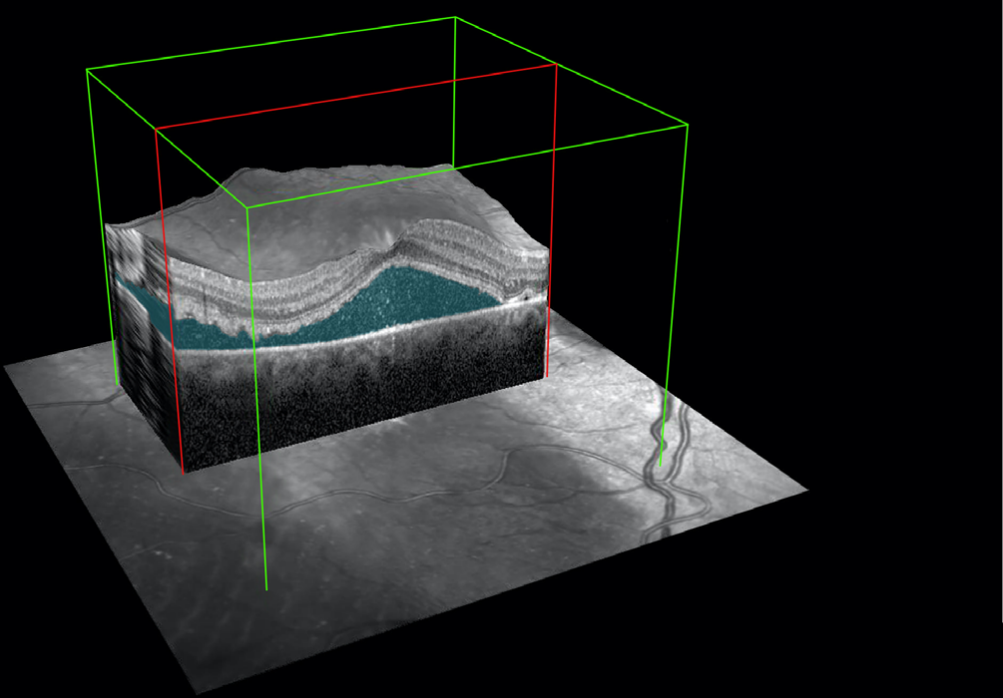
A spectral‐domain optical coherence tomography (SD‐OCT) image of an eye with macula‐off detachment. We defined retinal microstructures using the dense volume protocol with horizontal 20° × 20°, 25‐line raster scans of the macula and obtained automated segmentation and volume using the SD‐OCT software (Heidelberg Engineering, Inc., Heidelberg, Germany). The submacular fluid volume comprises the volume and detachment height, the distance between the basal membrane and outer border of the retinal pigment epithelium

We selected a retinal thickness map to quantify the SMFV of retinal detachment, which comprised nine Early Treatment Diabetic Retinopathy Study macular subfields (ETDRS) and a centre point with a 6‐mm diameter through the fovea. The built‐in segmentation algorithm measured the average thickness and average volume of each zone and the centre. We measured SMFV (mm^3^) between the basal membrane and outer border of the retinal pigment epithelium (RPE) (Figure 1) of the central 6‐mm diameter defined by the ETDRS grid. The foveal detachment height (mm) was measured between the basal membrane and the outer border of the RPE at the central 1‐mm diameter through the fovea. The CMT was defined as the average thickness of the central 1‐mm diameter through the fovea measured between the outer border of the RPE and the inner border of the internal limiting membrane.

The software calibrated a height up to 1.635 mm and calculate cases with an SMFV up to 20.6 mm^3^. Thus, we imputed a conservative value of 1.635 mm and 20.6 mm^3^ for each value exceeding the threshold to perform calculations in cases with increased detachment or SMFV.

### 2.4 Surgical procedures

Vitreoretinal surgery was conducted at a tertiary medical centre based in the federal state of Tyrol. The surgeries were performed under peribulbar or general anaesthesia. All cases were treated with standard small gauge (23G, 25G, 27G) pars plana vitrectomy (PPV) with the use of fluid‐air exchange with gas (C_3_F_8_) as an endo‐tamponade. Heavy liquids (perfluorocarbon) and retinotomies for internal drainage were optional elements of PPV procedures.

### 2.5 Statistical analyses

Demographic data and baseline findings are presented as the number of patients with percentages, while continuously distributed data are reported with their mean and SD. We used the Kolmogorov‐Smirnov test to determine whether data were normally distributed, and unpaired sample *t*‐tests and analysis of variance were used to assess normally distributed data. We compared non‐normally distributed data using the Mann‐Whitney *U*‐test and categorical data using the Chi‐squared and Fisher's exact tests.

We also calculated Pearson's correlation or Spearman's rank correlation coefficient to analyse correlations among parameters. Furthermore, we conducted univariate and multivariate regression analyses to determine the potential confounders of BCVA at 12 months after macula‐off RRD surgery. All factors with a *P*‐value of <.2 on the univariate analysis and a variance inflation factor of <5 through the BCVA at the 12‐month follow‐up visit were selected as independent variables for linear regression analyses performed to assess visual outcomes at 3‐, 6‐ and 12‐month follow‐up visits. Binary logistic regression was used to determine the odds ratio of impaired visual outcome in SMFV subgroups. Values of *P* < .05 were considered significant. Statistical analyses were performed using SPSS 25 Statistics software (IBM Corp., Armonk, New York).

## 3 RESULTS

### 3.1 Patient characteristics

A total of 127 eyes met our inclusion criteria for retrospective analysis. The mean patient age at the time of presentation at our clinic was 63 ± 14 years. Fifty‐six (44%) patients were phakic at the time of RRD diagnosis. Three (2%) patients underwent a lens‐sparing vitrectomy, none of whom showed significant lens opacities at the follow‐up examinations. Further characteristics are listed in detail in Table 1.

**TABLE 1:** Demographics and baseline characteristics of the patients with primary macula‐involving rhegmatogenous retinal detachment

Baseline characteristics	*n*, Mean, or median (%, SD, or IQR)
Age (years)	63 (14)
Sex (M/F)	89 (70)/38 (30)
Eyes (OD/OS)	65 (51)/62 (49)
Myopia (≥26.5 mm)	8 (6)
Pseudophakic	53 (42)
PPV	127 (100)
23 G	54 (43)
25 G	69 (54)
27 G	4 (3)
Retinal tears	1 (1–3)
Involvement of quadrants	
Superotemporal	75 (59)
Superonasal	58 (46)
Inferiornasal	76 (60)
Inferotemporal	100 (79)
Extent of detachment	6 (5‐7)
<3 hours	15 (12)
4‐6 hours	74 (58)
6‐9 hours	28 (22)
>9 hours	10 (8)
PVR	43 (34)
Grade A	26 (21)
Grade B	5 (4)
Grade C	12 (9)
Macula‐off duration (days)	3 (1‐6)
≤3 days	75 (59)
3‐7 days	31 (24)
7‐14 days	21 (16)
Baseline BCVA	1.3 (1.0‐1.7)
CMT (mm)	0.32 (0.24‐0.48)
Foveal detachment height (mm)	1.024 (0.667‐1.635)
SMFV (mm^3^)	11.2 (5.2‐20.6)

Abbreviation: BCVA, best‐corrected visual acuity; CMT, central macular thickness; IQR, interquartile range; PPV, pars plana vitrectomy; PVR, proliferative vitreoretinopathy; SMFV, submacular fluid volume.

Preoperative SMFV was significantly correlated with retinal detachment extent (*R* = 0.238; *P* = .015), preoperative BCVA (*R* = 0.557; *P* < .001), CMT (*R* = 0.808; *P* < .001). There was no correlation between SMFV and macula‐off duration (*R* = 0.118; *P* = .320).

### 3.2 Factors influencing visual outcomes

Table 2 shows potential confounding factors that may interfere with FVA 12 months after surgical interventions for macula‐off RRDs. In the univariate analysis, macula‐off duration (*P* = .005), foveal detachment height (*P* < .001), CMT (*P* = .011), lower BCVA (*P* = .002) at baseline and central SMFV *(P <* .001) were significantly associated with lower visual outcomes 12 months after foveal reattachment. After creating a linear regression model, central SMFV (ß = 0.013; *P* = .005) and a macula‐off duration (ß = 0.020; *P* = .033) were determined to be the factors that principally influenced FVA 12‐months post‐RRD surgery.

**TABLE 2:** Preoperative factors influencing best‐corrected visual acuity 12 months after macula‐involving primary rhegmatogenous retinal detachment surgery

	Univariate analysis		Multivariate analysis	
Variables	β (SE)	*P*	β (SE)	*P*
Age (years)	0.000 (0.003)	.951	—	—
Sex	−0.05 (0.067)	.940	—	—
Myopia (≥26.5 mm)	0.028 (0.148)	.850	—	—
Pseudophakic	0.037 (0.061)	.547	—	—
PPV			—	—
23 G	Reference		—	—
25 G	0.000 (0.062)	.994	—	—
27 G	−0.113 (0.177)	.523	—	—
Number of retinal tears	0.019 (0.020)	.341	—	—
Involvement of quadrants				
Superotemporal	0.139 (0.061)	.024*	0.066 (0.069)	.344	
Superonasal	0.074 (0.061)	.224	—	—	
Inferonasal	−0.063 (0.060)	.297	—	—	
Inferotemporal	−0.080 (0.074)	.283	—	—	
Extent of detachment	0.013 (0.013)	.335	—	—	
<8 hours	Reference		Reference		
>8 hours	0.111 (0.077)	.152	−0.001 (0.078)	.990	
PVR					
None	Reference		Reference		
Grade A	0.130 (0.078)	.096	0.077 (0.079)	.335	
Grade B	0.020 (0.157)	.896	0.037 (0.154)	.809	
Grade C	0.035 (0.105)	.736	0.113 (0.108)	.299	
Macula‐off duration (days)	0.021 (0.007)	.005*	0.020 (0.009)	.033*	
1‐3 days	Reference		Reference		
3‐7 days	−0.130 (0.093)	.163	−0.097 (0.096)	.315	
7‐14 days	0.137 (0.080)	.090	0.171 (0.085)	.047^*^	
Baseline BCVA	0.182 (0.057)	.002^*^		—^a^	
CMT (mm)	0.720 (0.278)	.011^*^		—^a^	
Foveal detachment height (mm)	0.224 (0.059)	<.001^*^		—^a^	
SMFV (mm^3^)	0.016 (0.005)	<.001^*^	0.013 (0.005)	.005^*^	

Abbreviation: BCVA, best‐corrected visual acuity; CMT, central macular thickness; PPV, pars plana vitrectomy; PVR, proliferative vitreoretinopathy; SE, spherical equivalent; SMFV, submacular fluid volume.

^a^ Excluded from multivariate analysis because of a high variance inflation factor (>5).

* Statistical significance (*P* < .05).

Factors that correlated with visual outcomes 12 months after surgery with a *P*‐value <.2 in the univariate analysis were included in the multivariate analysis to scrutinise their effect on visual outcomes at 3‐ and 6‐month follow‐up. Multivariate analysis of the 3‐month follow‐up revealed that detachment for over 8 hours was the only factor associated with reduced BCVA (ß = 0.235; *P* = .046). SMFV (ß = 0.010; *P* = .025) and macula‐off duration (ß = 0.019; *P* = .022) were significantly associated with a reduced BCVA 6‐month post‐surgery (Table 3).

**TABLE 3:** Multivariate analysis of potential preoperative factors influencing best‐corrected visual acuity 3, 6 and 12 months after macula‐involving primary rhegmatogenous retinal detachment surgery

Variables	3 months		6 months		12 months	
	β (SE)	*P*	β (SE)	*P*	β (SE)	*P*
Involvement of superotemporal quadrant	0.114 (0.091)	0.214	0.053 (0.069)	.437	0.066 (0.069)	.344
Extent of detachment						
≤8 hours	Reference		Reference		Reference	
≥8 hours	0.235 (0.122)	0.046*	0.013 (0.078)	.863	−0.001 (0.078)	.990
PVR						
None	Reference		Reference		Reference	
Grade A	0.145 (0.116)	0.217	0.072 (0.079)	.363	0.077 (0.079)	.335
Grade B	0.165 (0.206)	0.428	0.004 (0.153)	.981	0.037 (0.154)	.809
Grade C	0.056 (0.143)	0.697	0.043 (0.107)	.688	0.113 (0.108)	.299
Macula‐off duration (days)	0.004 (0.013)	0.773	0.019 (0.008)	.022*	0.020 (0.009)	.033*
1‐3 days	Reference		Reference		Reference	
3‐7 days	−0.054 (0.131)	0.682	−0.126 (0.095)	.189	−0.097 (0.096)	.315
7‐14 days	0.067 (0.121)	0.582	0.155 (0.084)	.069	0.171 (0.085)	.047*
SMFV (mm^3^)	0.000 (0.006)	0.977	0.010 (0.005)	.025*	0.013 (0.005)	.005*

Abbreviation: PVR, proliferative vitreoretinopathy; SE, spherical equivalent; SMFV, submacular fluid volume.

^*^ Statistical significance (*P* < .05).

### 3.3 Role of SMFV in predicting visual outcome

**FIGURE 2:**
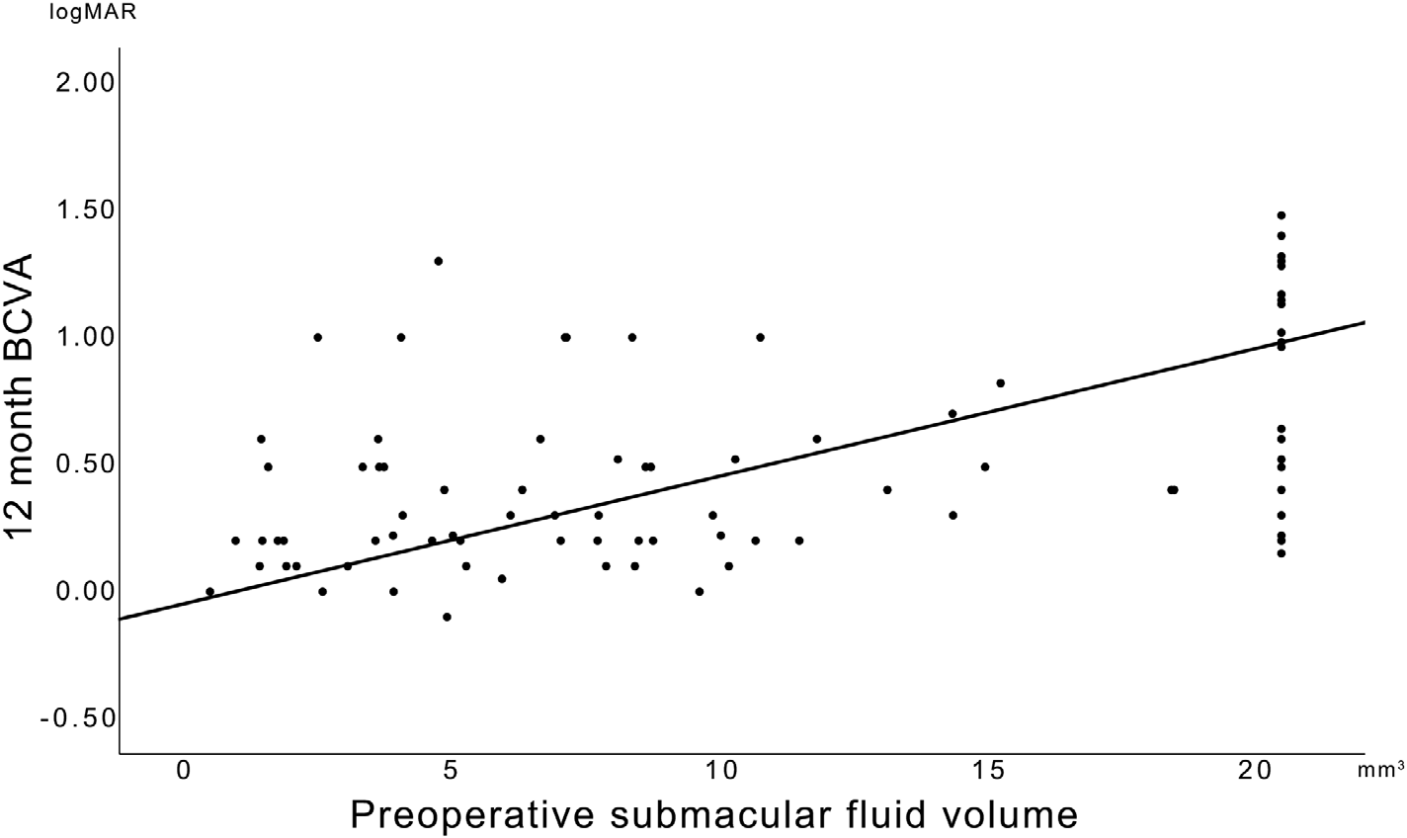
Correlation between preoperative submacular fluid volume and best‐corrected visual acuity (BCVA) given as the logarithm of the minimum angle of resolution (logMAR) 12 months after the surgical management of macula involving primary rhegmatogenous retinal detachment

Negative correlations were found between preoperative SMFV and 12‐month postoperative VA (0.41 ± 0.34; *r* = 0.311; *P* = .001) (Figure 2). Patients with a high SMFVs had a BCVA logMAR of 0.166 ± 0.066 (*P* = .006) and 0.196 ± 0.075 (*P* = .001) worse than patients with low SMFVs 6‐ and 12‐month post‐surgery, respectively. When the binary logistic regression was adjusted based on the extent of retinal detachment, detached quadrants, PVR and macula‐off duration, we found that patients with a low SMFV (≤9.0 mm^3^; *n* = 64, 50%) had significantly better visual outcomes than patients with a high SMFV (9.0‐20.6 mm^3^) at 6‐months [(*P* = .042; 95% confidence interval (CI) = 1.057‐24.894] and 12‐month post‐surgery [(*P* = .018; 95% CI = 1.438‐44.514). Patients with a high SMFVs were predicted to have poor visual outcomes at rates 5.1 times higher 6‐months post‐surgery and 8.0 times higher 12‐month post‐surgery than those with low SMFVs.

**FIGURE 3:**
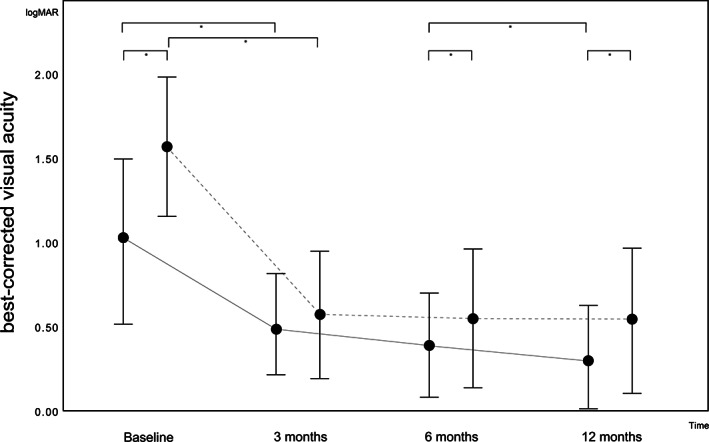
Best‐corrected visual acuity given as logarithm of the minimum angle of resolution (logMAR) with SD in patients with low (≤9.0 mm^3^; continuous line) or high (9.0‐20.6 mm^3^; dashed line) submacular fluid volume adjusted to macula‐off duration and extent of retinal detachment at baseline and 3, 6 and 12 months postoperatively. *Statistical significance (*P* < .05) in the multivariate analysis was adjusted for macula‐off duration and extent of retinal detachment

The BCVA significantly improved from baseline (1.09 ± 0.42) to 12‐month (0.31 ± 0.28; *P* < .001) follow‐up in the low SMFV group [3 months (0.43 ± 0.30) to 6 months (0.37 ± 0.31) *P* = .102; 6‐12 months *P =* .011] and from baseline (1.54 ± 0.40) to 12‐month (0.52 ± 0.36; *P* < .001) follow‐up in the high SMFV group [3 months (0.52 ± 0.38) to 6 months (0.53 ± 0.35; *P* = .642); 6 to 12 months *P =* 0.735] (see Figure 3).

## 4 DISCUSSION

RRD is a vision‐threatening disease that requires surgical intervention to prevent permanent functional damage to the neurosensory layer. In cases of macula‐off RRD, central vision prognosis remains difficult to estimate. In this study, we employed a fully automatic segmentation algorithm software for SD‐OCT to quantify SMFV and evaluate its effect on the visual outcomes at 3, 6 and 12 months after surgical treatment for macula‐off RRD. We found a significant negative correlation between preoperative SMFV and 12‐month postoperative VA. Furthermore, in a comprehensive analysis, we revealed that the SMFV and duration of macular detachment were independent key risk factors for reduced visual recovery. Patients with a low SMFV showed steady visual improvement throughout 12 months of follow‐up. Compared to the high SMFV group, the low SMFV had significantly better visual outcomes 6‐ and 12‐month post‐surgery. Thus, our results suggest that patients presenting with SMFV values below 9.0 mm^3^ have an 8.0 times greater chance for a better visual recovery after surgical intervention. This value may be used as a predictive factor for visual outcomes when counselling patients.

A long macula‐off duration is well known to be associated with a worse visual outcome. Several studies have correlated the effect of time on macula‐off RRDs and have shown varying visual outcomes after surgical repair. ^3^, ^4^, ^5^, ^13^, ^16^ In our study, patients who received surgical treatment more than 7 days after the onset of central vision loss had a worse visual outcome than did patients who underwent earlier reattachment. Although our conclusion is in line with numerous previous reports,^6^, ^7^, ^8^, ^16^ some other studies asserted that very early treatment, within 3 days of central vision loss, should be performed to preserve visual outcomes in patients treated for macula‐off RRDs.^3^, ^5^ From a patient perspective, however, the precise perception of central vision loss seems to be difficult. While Ricker et al^17^ reported that 25% of patients did not recognise macula involving detachment and central vision loss, Ng et al^18^ found that 15% to 40% with actual macula on detachment gave answers consistent with the presence of a detached macula. These findings indicate that there is great potential for variation in subjective symptoms between individuals. Therefore, the accurate dating of foveal detachment is a limitation faced by all studies that address surgical timing of macula‐off RRD. The involvement of further predictive factors provides an additional explanation for the broad range of visual outcomes produced following surgical treatment of macula‐off RRD. Therefore, it might be beneficial to perform OCT preoperatively in all cases to assess and confirm the foveal status. This allows for a straightforward quantification of objective factors influencing visual outcomes besides the limited, rather subjective, clinical onset of vision loss.

Numerous previous studies have investigated the influence of other various factors associated with reduced FVA. Visual acuity at baseline, age, retinal detachment height, the extent of retinal detachment and method of operation are related to reduced visual recovery.^11^, ^16^, ^19^ However, some studies did not conduct a multivariate analysis or adjust OCT findings to their multivariate analysis.^16^, ^19^, ^20^, ^21^, ^22^, ^23^ In this study, we found that as per the univariate analysis, there was a significant correlation between FVA and SMFV, CMT, BCVA at baseline, foveal detachment height, macula‐off duration. However, after performing and adjusting the multivariate analysis, only SMFV and macula‐off duration were associated with worsened visual outcomes 12 months post‐surgery. The extent of retinal detachment appears to have a short‐term effect on visual recovery 3 months after surgery but did not affect final visual outcome.

Regarding the influence of the extent of retinal detachment, a previous study reported that a more advanced retinal detachment is correlated with a significant increase of interleukin‐8 (IL‐8) and transforming growth factor beta‐3 (TGFβ‐3) in the vitreous humour, which indicates an inflammatory reaction due to an underlying apoptotic process in the detached outer retinal layer.^24^ The same study also reported a significant correlation between tissue inhibitor metalloproteinase‐1 (TIMP‐1) and retinal detachment height. TIMP‐1 is known to suppress programmed cell death. Since foveal detachment height in RRD cases is associated with reduced visual recovery due to increased cell apoptosis,^12^, ^25^, ^26^ a counterregulatory response of protective mediators is likely to occur.

Animal studies also support the correlation between detachment height in RRD cases and outer retinal damage. Disruption and loss of photoreceptors and 20% thinning of the outer nuclear layer have been observed within 72 hours of detachment.^14^ The important role of nutrients and oxygen has been highlighted by reduced photoreceptor damage in the presence of hyperoxic conditions during retinal detachment in an animal model.^15^ Therefore, the authors of these two studies emphasised the importance of maintaining the oxygen supply from the choriocapillaris.^14^, ^15^ Damage to the outer neurosensory layer might be more extensive in increased detachment cases due to reduced diffusion of oxygen and essential nutrients. However, the effect of impaired oxygen diffusion cannot be caused by detachment height changes alone. The area of macular detachment and central SMFV must also be considered. Therefore, we propose the use of SMFV rather than detachment height in future studies to minimise this potential bias.

There are several limitations to our study. First, this study is retrospective and used data collected in a structured electronic register to monitor quality and efficacy indicators for vitreoretinal surgery in patients with primary RRD at one tertiary referral centre. Second, the effect of SMFV and central height of detachment on the visual outcomes may have been underestimated because heights over 1.635 mm were recorded as 1.635 mm and SMFVs >20.6 mm^3^ were recorded as 20.6 mm^3^ due to technical limitations of SD‐OCT. In these cases, the long wavelength used by swept source (SS)‐OCT devices could provide the potential of deeper axial imaging ranges.^27^ However, deeper penetration into the choroid with the longer wavelength comes at the cost of reduced axial resolution and contrast of the retinal layers,^28^ and might result in less reliable retinal segmentation and consecutive impairment of analytics for automated fluid volumetric quantification. Nonetheless, future investigations could compare SD‐OCT and SS‐OCT for the automated quantification of SMFV to further elucidate the effect of even higher SMFV on the final visual outcome. Third, in our study group, phacovitrectomy was performed in almost all cases with phakic RRD following intern standards that recommend a combined procedure to prevent risks caused by a sequential cataract surgery (such as endophthalmitis, increased capsular rupture rate in vitrectomized eyes).^29^ Recent findings by Tan et al^30^ confirmed that visual outcomes should not be affected by phacovitrectomy compared to single PPV in the management of phakic eyes with retinal detachment. None of the four cases that were performed as lens‐sparing vitrectomy in our study cohort showed lens opacities at the follow up examinations.

A strength of the study was that SD‐OCT was performed in all cases to assess and confirm the foveal involvement of the RRD. We used a segmentation algorithm that facilitated the exact volumetric quantification of submacular fluid in patients undergoing surgical intervention for primary RRD. To the best of our knowledge, the present study is the first that measured SMFV using OCT volumetric quantification in patients with macula‐off primary RRD. The findings of our study could help clinicians understand the extent to which SMFV affects visual recovery after surgical management of macula‐involved primary RRD. Future studies should investigate submacular volume rather than retinal detachment height as a more accurate parameter.

In conclusion, this study identified a negative correlation of preoperative SMFV and final visual outcome. Macula‐off duration and SMFV were key independent risk factors for an impaired visual outcome following surgical intervention for macula‐off RRD. While patients with preoperative SMFV <9.0 mm^3^ experienced a steady improvement of VA up to 12 months post‐surgery, patients with high preoperative SMFV of 9.0 mm^3^ had no VA improvement more than 3 months after surgery. Patients presenting with low SMFV had an 8.0 times greater chance of visual recovery after surgical reattachment.

## CONFLICT OF INTEREST

None declared.

## Data Availability

The data that support the findings of this study are available from the corresponding author, upon reasonable request.
